# Experience of loss and grief among people from Germany who have lost their relatives during the pandemic: the impact of healthcare professionals' support

**DOI:** 10.3389/fpubh.2023.1230198

**Published:** 2023-08-16

**Authors:** Arndt Büssing, Klaus Baumann

**Affiliations:** ^1^Professorship Quality of Life, Spirituality and Coping, Faculty of Health, Witten/Herdecke University, Herdecke, Germany; ^2^Caritas Science and Christian Social Work, Faculty of Theology, Albert-Ludwig-University, Freiburg, Germany

**Keywords:** bereavement, grief, loss, pandemic, health care provider, wellbeing

## Abstract

**Background:**

Due to public restrictions during the early stages of the COVID-19 pandemic, many people were unable to visit and bid a proper farewell to their dying loved ones. This study aimed to address the loss-oriented aspects of grief and bereavement of relatives and relate these to the support they may have received from their dying relative's caring professionals.

**Materials and methods:**

People from Germany who experienced bereavement during the COVID-19 pandemic were enrolled in a cross-sectional study between July 2021 and May 2022, using standardized questionnaires (i.e., ICG, Inventory of Complicated Grief; BGL, Burdened by Grief and Loss scale; WHO-5, WHO-Five Wellbeing Index; and 5NRS, perception of burden related to the pandemic).

**Results:**

Most participants (*n* = 196) had the opportunity to visit their relatives before death (59%). When this was not possible, being burdened by grief and loss was significantly higher (Eta^2^ = 0.153), while this had no significant influence on complicated grief or psychological wellbeing. Furthermore, 34% of participants felt well-supported by the treatment/care team. Their own support was moderately correlated with BGL scores (*r* = −0.38) and marginally with ICG scores (*r* = −15). Regression analyses showed that complicated grief symptoms as the dependent variable were predicted by (low) psychological wellbeing, relational status, and the perception of COVID-19-related burden (*R*^2^ = 0.70). In contrast, BGL as the dependent variable can be best explained by the perception of emotional affections because of restricted visits shortly before their death, by the (short) duration of visits before death, and by the relational status (*R*^2^ = 0.53). Although both were interconnected (*r* = 0.44), their predictor pattern was different.

**Conclusion:**

Being able to visit dying relatives was important for the mourning and bereavement processes. This emotional aspect was more relevant to the normal, non-pathological grief and loss processes than to complicated grief processes. Support from their dying relatives' treatment/care team was highly relevant to the mourning process, but the visiting relatives often lacked information about additional resources such as psychologists or pastoral care professionals or had limited access to them.

## 1. Introduction

Due to public restrictions during the early stages of the COVID-19 pandemic, many people were unable to visit and bid a proper farewell to their dying loved ones. Aware of this dramatic situation, even the federal president of Germany, Frank-Walter Steinmeier, held a central event in memory of the victims of the COVID-19 pandemic in Berlin on 18 April 2021, giving voice to the harm and suffering experienced by the deceased and their bereaved relatives, families, and friends (1). Due to quarantine protocols, most nursing/caring homes, hospitals, and hospices were closed to visitors. Terminally ill patients were isolated from others, including their relatives, and were often dying alone. In the best cases, visits—with protective masks and clothing—were reduced in number or permitted at least in the final phases of dying. This had an impact on both the dying people and their visitors, who were used to living with their relatives for decades in close relationships. Due to the physical distancing and social isolation regulations, the bereavement rituals were restricted as well, and even visits to public funerals were held remotely (2, 3). What impact did these pandemic-related restrictions have on the experience of loss and grief among people who lost their relatives during the pandemic?

### 1.1. Difficult bereavement processes because of pandemic-related restrictions

Evidently, the pandemic “has disrupted grief experiences of bereaved relatives and altered accustomed ways of coping with loss” (4). Social isolation resulted in a lack of physical and emotional support for the dying by family and friends—who themselves felt lonely and disrupted (5). The review by Stroebe and Schut on bereavement in times of the COVID-19 pandemic underlined various emotional reactions of bereaved people toward governmental institutions on the one hand and healthcare professionals on the other, which may aggravate prolonged grief (5). In their overview, van Schaik et al. (4) also addressed the “effect of absence during final moments” and the “lack of involvement in the caring process.” Goveas and Shear (6) described the risk factors of prolonged grief processes. A relevant risk factor of poor bereavement was related to pandemic-related restrictions, particularly the “need to isolate patients to control the spread of COVID-19” (7). Isolation of patients in intensive care units was a relevant stressor for patients' relatives even before the pandemic (8), and it has been related to complicated grief, post-traumatic stress, and depressive symptoms, summed up as post-traumatic stress disorder symptoms (9).

Selman et al. (7) described recommendations for hospital clinicians to support relatives' bereavement processes. These additional tasks (given as advice) could be regarded as an additional burden, as most of the hospital staff had to deal with increasing stress and work overload and, simultaneously, had to cope with their own fears and worries (10). They were not only caring for the dying (both with COVID-19 infection and without) but also for their patients' grieving relatives (7, 11). Consequently, the risk of compassion fatigue and burnout among healthcare professionals was high, which was aggravated by the pandemic situation (12). Unresolved grief, as measured with the Inventory of Complicated Grief (ICG), was reported not only by patients' relatives but also by nursing staff during the pandemic (13). In their study from Iran, a large proportion of nurses were reported to experience complicated grief [particularly women and nurses working in COVID-19 intensive care units (ICUs)], and this was ascribed to their “frequent exposure to patients' deaths” (13). Nevertheless, findings from ICU staff members from Iran who lost their own family members during the pandemic showed that they had complex grief processes, but that they paid “more attention to their patients and the people who were accompanying the patient” (14).

### 1.2. Different courses of grief processes because of pandemic-related restrictions

Loss of a beloved person usually results in grief and bereavement processes associated with feelings of sadness, guilt, and anger, among others (15). These normal, non-pathological processes are mostly self-limiting as people learn to cope with their loss. The necessary mourning process requires time. However, in some situations, people experience prolonged phases of grief, which can be regarded as a disorder. The International Classification of Diseases (ICD-11) uses the diagnosis “Prolonged Grief Disorder” (PGD), which is defined as a “persistent and pervasive longing for the deceased” or a “preoccupation with the deceased” (more than 6 months) accompanied by intense emotional pain (15–17). An additional concept is “complicated grief” (CG), which points in a similar direction but uses other diagnostic criteria (18, 19).

Given the assumption that COVID-19-related circumstances may result in prolonged grief reactions (20), it is important to describe the loss characteristics and circumstances, as well as the grief levels of people before and within the pandemic. Eisma and Tamminga (21) confirmed that the different grief levels (which were higher in COVID-19 deaths than in natural deaths) can be explained by the “expectedness of the death and the inability to say goodbye.” They argued that people infected with COVID-19 (who were finally dying) were more often treated in ICUs, which means that they had reduced accessibility to visitors and that COVID-19 deaths were followed by “altered funeral services,” which affected grief rituals (21). However, there is little empirical evidence on this topic (22). A large fraction of people whose relatives were dying because of a COVID-19 infection reportedly showed signs of clinically relevant dysfunctional grief (23). In that study, participants' functional impairment was not related to sociodemographic data or time since loss but to COVID-19, the need for professional help, and the closeness of the relationship. However, during the pandemic, people were also dying without a COVID-19 infection, and their relatives were affected by the restrictions as well.

PGD was high (67.5%) in a self-selected sample who lost a significant other between October 2020 and July 2021 (24). In the study, the time since death and the closeness of the relationship were relevant factors. Even before the pandemic, Kentish-Barnes et al. (9) showed that, after the death of a relative in an ICU, which is characterized by restricted access to visits, 52% of people had symptoms of CG. Downar et al. (25) compared the relatives of those who died of COVID-19 or other conditions during the first wave of the pandemic or of those who died before its onset. In their study, 29% of family members had severe grief symptoms, and the prevalence of grief was similar between both cohorts studied before and during the pandemic. They found no association between the severity of grief and sociodemographic factors, physical presence, or relationship with the deceased (25). Thus, any deadly loss during pandemic-related restrictions might have been painful for the bereaved; thus, the conditions of parting and the support they received were crucial. Qualitative analyses of the “Bereavement Welfare Hub” in London showed that good communication between the ward members and the patient, the ability to visit and be present at death, family and community support, bereavement support, and death rituals and customs positively influenced the bereavement experiences and grief status of those who sought support during the first wave of the pandemic (26). Thönnes and Noll-Houssong (27) highlighted that it has not yet been possible to validate the assumed increased prevalence of persistent mourning disorders. There remains a dearth of empirical studies taking into account the bereaved relatives who experienced the loss of a beloved person due to or during the pandemic (21, 22, 28, 29). Chen and Tang (22) concluded in their study of 422 Chinese participants who were bereaved due to the COVID-19 pandemic that serious attention needs to be paid to the mental health issues of these people. Almost 70% of this group had a moderate-combined or high-combined symptom profile of prolonged grief. The researchers also emphasized the need for special care for those who lost someone younger, those who lost a partner, or those who shared a close relationship with the deceased.

### 1.3. Aim of this study

In our study, we thus focused on the loss-oriented aspects of bereavement and grief of relatives, as well as the support they may have received from caring professionals (i.e., the hospital, nursing home, and hospice). We differentiated CG and the difficulties of letting go and the perception of loss (because of pandemic-related restrictions) among people who were bereaved during the COVID-19 pandemic. Owing to the relevance of the positive influence of good communication and psychological support by the ward, as well as the respective bereavement support (26), we also addressed support satisfaction and the wish for psychological or pastoral accompaniment. We studied these perceptions in relation to relatives' satisfaction with the support they received from the treatment/care team of their dying relatives and the possibility of visiting their relatives shortly before their death. We further addressed the relational status, as some studies reported it to be of relevance (24), while others did not (25).

**Figure 1:**
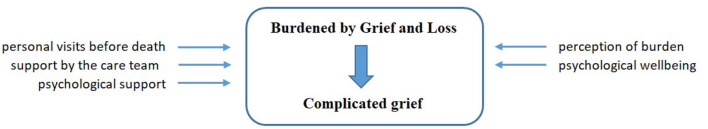
Theoretical model of selected variables with an influence on grief processes. These topics are addressed by the specific measures described below.

According to the findings from the literature described above, we assumed that the intensity of normal, non-pathological grief processes (in terms of the burden of grief and loss) is inversely related to both the possibility of visiting the dying relatives (9, 21) and the support received from the care team (23, 26). In contrast, CG processes depended on several other factors, including feelings of sadness, guilt, social isolation/loneliness, and disruption during the pandemic (5, 9), which we regarded as the perception of burden and psychological wellbeing. Thus, CG processes seem to be less dependent on the possibility of visiting dying relatives and the support received from the care team (Figure 1).

## 2. Materials and methods

Reporting of methods and data in this cross-sectional survey followed the STROBE guideline of cross-sectional studies, where possible.

### 2.1. Recruitment of participants

People who were bereaved during the COVID-19 pandemic were invited to participate in an anonymous online survey (via LimeSurvey) between July 2021 and May 2022. The archbishop's pastoral office in Freiburg and the Palliative Care Forum Freiburg drew the attention of relevant people from pastoral care and bereavement support to the research project and asked them to invite mourners to participate. In addition, information was sent to the network of cooperation partners so that participation was also advertised in a snowball system.

As the topic was sensitive, we expected that some participants would again be confronted with the experience of loss. We chose the method of an anonymous survey to give participants the emotional distance they might need; in case they needed to talk to someone about their experiences, the pastoral office team was available, and additional addresses for support were also provided with the survey. There were no incentives for participation. Thus, the participants decided by themselves whether they wanted to participate in the study and to which parts of the questionnaire they wanted to respond to. We inferred that the topic was burdensome for several of the participants who were initially interested and responded only to the first parts of the survey (“starters”). We stopped the survey after 10 months of repeated snowball initiatives when no new responses were recorded. We had no exclusion criteria; instead, we invited all those who have lost a relative during the pandemic to participate.

No identifying details were requested from the participants nor were their IP addresses stored to ensure that their privacy was fully protected from third parties. Further information on data protection was made available on the survey page, where participants had to click on the “consent to participate” box. They were also advised on pastoral care and bereavement support, which they could seek when needed. The study was positively voted for by the ethics committee of the University of Witten/Herdecke (S-122/2021).

### 2.2. Measures

The questionnaire included sociodemographic data of the participants, information about the deceased and the farewell process, and the resulting need for conversations, talks, and support, among others, which might give relief. The psychometric instruments that were used in the German language version are described in the following sections.

#### 2.2.1. Complicated grief

To address prolonged or CG situations, we used the 19-item ICG by Prigerson et al. (30). The German language version (ICG-D) has good internal consistency in the validation sample (Cronbach's alpha = 0.87) (31) and high consistency in this sample (Cronbach's alpha = 0.92). Representative items are “I feel I cannot accept the death of the person who died,” “I feel drawn to places and things associated with the person who died,” “I feel that life is empty without the person who died,” “I hear the voice of the person who died speak to me,” and “I feel that it is unfair that I should live when this person died.” It scores from never (0), rarely (1), sometimes (2), and often (3) to always (4).

In the German validation study, the mean ICG-D sum score was 36.3 ± 13.2, with a range of 5–66 (31). Prigerson et al. (30) suggested a cut-off level of 25, while Shear et al. (19) used a much higher score of 30. However, in our study, with a mean score of 21 ± 12, one SD above the mean would be 33. As more participants with WHO-5 scores <13 had a mean ICG score of 28 ± 13, we used 33 as a safer cut-off score. Accordingly, we suggested scores <8 as no relevant grief reaction, and scores between 8 and 33 as “normal” grief reactions.

#### 2.2.2. Burdened by Grief and Loss

Perceived difficulties in parting from relatives and the perception of loss because of pandemic-related restrictions were addressed with the 9-item German language scale “Schwierige Abschiednahme und Verlustempfinden” (SAVE), which can be translated as being “Burdened by Grief and Loss” (BGL).

The nine items address feelings of sadness and guilt about the fact that it was not possible to be with the relative during their last days because of pandemic-related restrictions:

“I couldn't spend enough time with my relatives due to the contact restrictions,” “When I think of not being able to adequately accompany my [relative] in his dying, I am still overcome with great sadness,” “I just can't deal with the fact that I wasn't able to adequately accompany my [relative] in his/her dying,” “I still feel terrible that I hardly had a chance to say a proper goodbye to my [relative],” “It hurts so much that my [relative] had to die alone like that,” “I feel it is unfair that I could not visit my dying [relative] regularly,” “I have been with my [relative] all my life, but I was denied that during the Corona pandemic,” “I feel guilty that I could not be there appropriately for my [relative],” “In regard of the death of my [relative], I am still angry that I was not able to visit him as often as I should have.”

They are scored on a 5-point Likert scale ranging from does not apply at all (0), does not really apply (1), indifference/neither yes nor no (2), and applies quite well (3) to definitely applies (4). The respective items of this new scale have excellent internal consistency (Cronbach's alpha = 0.94). The scale is unidimensional and explains 67% of the variance; item loadings range from 0.70 (“I couldn't spend enough time with my relatives due to the contact restrictions”) to 0.89 [“I just can't deal with the fact that I wasn't able to adequately accompany my (relative) in his/her dying”]. The BGL scores correlate strongly with psychological wellbeing (WHO-5: *r* = −0.54) and moderately with CG (ICG-D: *r* = 0.47).

As the cut-off, we used one SD above the mean (18 ± 11). This means that scores > 29 indicate a higher burden of grief, scores <7 indicate no relevant burden, and scores between 7 and 29 indicate a moderate burden of grief. Participants with low psychological wellbeing (WHO-5 scores <13) had BGL scores of 23.5 ± 9.8.

#### 2.2.3. Psychological wellbeing

Psychological wellbeing was measured with the WHO-Five Wellbeing Index (WHO-5) (32). It uses five items such as “I have felt cheerful and in good spirits” or “My daily life has been filled with things that interest me.” The frequency of these experiences is scored from at no time (0) to all of the times (5). In this study, we reported the sum scores ranging from 0 to 25 and scores <13 indicated low wellbeing or even depressive states. In this sample, the scale had excellent internal consistency (Cronbach's alpha = 0.93).

#### 2.2.4. Perception of burden

Perceived restrictions of daily life (e.g., being under pressure and stressed, being anxious and insecure, being lonely and socially isolated, and being burdened by a difficult financial-economic situation) because of the COVID-19 pandemic were measured with five numeric rating scales (5NRS), ranging from 0 (not at all) to 100 (very strong) (33). These five variables could be combined to the factor COVID-19-related burden, which had good internal consistency in the validation sample (Cronbach's alpha = 0.80) and in this sample (Cronbach's alpha = 0.78). Scores ranged from 0 (not at all) to 100 (extremely strong).

#### 2.2.5. Satisfaction with care support

Two items addressed satisfaction with the support received by their relatives and themselves from the treatment or care team: “I felt emotionally well-supported by the treatment/care team despite the difficult circumstances” and “I felt well-supported by the treatment/care team.” These perceptions were scored as no, not at all (0), rather no (1), partly (2), rather yes (3), and yes, very well (4).

Participants' wish for additional pastoral or psychological accompaniment could be expressed with two items and a yes/don't know/no scoring.

#### 2.2.6. Possibility of visits shortly before the death of their relative

The possibility of visits shortly before death was answered by a yes or no question. When visits were possible, participants reported that it helped them grieve and that they found it emotionally helpful. However, when visits were not (or only rarely) possible, they reported experiencing emotional affection or expressed that they missed these visits, which hindered the mourning process. Four of these items referred either to the possibility of personal visits shortly before death (V1 “How much did the personal visits support you emotionally in the situation?” and V2 “Looking back, how helpful were the personal visits this for your grieving?”) or to restricted possibilities (V3 “How much did the visit restrictions emotionally affected you down in the situation?” and V4 “Looking back, how much did you miss the visits for your mourning?”). These statements were scored as not at all/hardly (0), rather less (1), indifference (2), rather more (3), and very much (4).

#### 2.2.7. Relational/generational status

The relational/generational status toward the dying person was categorized as parents (G1 generation), partner/brother or sister (G2 generation), or relatives/others.

Participants were also asked about their (emotional) relationship with the deceased. Relationships were categorized on a 5-point Likert scale as very distant (1), rather distant (2), neutral (partly partly) (3), rather close (4), and very close (5).

### 2.3. Statistical analyses

Descriptive statistics are presented for all relevant variables as frequencies for categorical variables and as means (± standard deviation, SD) for numerical variables. For the different subgroup analyses (i.e., gender, relational status, place of dying, visits at place), analyses of variance (ANOVA) were performed. The focus variables (grief, wellbeing, and burden) were further correlated with different indicators of the possibility of visits and their emotional reactions, as well as perceived support, using first-order correlations (Spearman rho). Linear regression analyses with a stepwise variable selection method based on probabilities (*p*-values) were performed to identify the best predictors of CG and those of being BGL as dependent variables. These analyses followed the assumptions described in the aim of the study. Missing data relevant to the scales were replaced (multiple imputations), while all other statements were taken as they were and were not replaced. All statistical procedures were computed with SPSS 28.0.

Given the exploratory nature of this study, we set a stricter significance level at a *p*-value of <0.01. With respect to classifying the strength of the observed correlations, we adjusted the thresholds to *r* > 0.5 as a strong correlation, an r between 0.3 and 0.5 as a moderate correlation, an *r* between 0.2 and 0.3 as a weak correlation, and an *r* < 0.2 as negligible or no correlation. For ANOVA, Eta^2^ values <0.06 were considered small effects, values between 0.06 and 0.14 were considered moderate, and those >0.14 were considered strong.

## 3. Results

### 3.1. Description of participants

A total of 236 people responded to the survey, but 40 of them gave no relevant information about themselves. Of the remaining (83% of those who started to respond), 35 completed the ICG questionnaire but not the other modules (their data were included in the current analysis), while 161 participants completed most of the questionnaire modules (68% of those who started to respond). These three groups did not differ significantly by gender, with non-responders being 6–8 years younger than others (*F* = 3.0; *p* = 0.053). In the following, datasets from 196 participants were taken into account.

A majority of the 196 participants were women (77%); they had a mean age of 47 ± 15 [19–86] years (Table 1). Owing to the recruitment routes, Catholics were disproportionately represented (56%); 19% were Protestants, 4% had other religious affiliations, and 21% had none. Furthermore, 50% identified as religious and spiritual (R+S+), 8% as religious but not spiritual (R+S-), 13% as not religious but spiritual (R-S+), and 29% as neither religious nor spiritual (R-S-).

**Table 1:** Description of the study sample (*n* = 196).

	***n***	%^*^	**Mean ±SD**
Age (years)	196		46.7 ± 14.6
**Gender**	196	100	
Women	151	77.0	
Men	44	22.4	
Diverse/no answer	1	0.5	
**Relational/generational status**	150	100	
Parents (G1)	54	36.0	
Partner/brother or sisters (G2)	25	16.7	
Relatives/others	71	47.3	
**Denomination**	196	100	
Catholics	110	56.1	
Protestants	37	18.9	
Other	8	4.1	
None	41	20.9	
**SpR self-categorization**			
R+S+	89	50.0	
R+S-	14	7.9	
R-S+	23	12.9	
R-S-	52	29.2	
**Place of dying**	150		
Hospital	85	56.7	
Nursing home hospice	30	20.0	
At home/Specialized Outpatient Palliative Care	35	23.3	
Months since death	147		10.2 ± 6.7
**Visits were possible**	136	100	
No	56	41.2	
Yes	80	58.8	
Number of visits	84		5.1 ± 7.6
Duration of visits (h)	129		7.3 ± 13.0

^*^% of responders.

Most of the participants' relatives died in a hospital (57%), 20% died in a nursing home or hospice, and 23% died at home. Additionally, 36% of the deceased people were fathers or mothers (G1 generation), 1% were children (*n* = 2; G3 generation), 17% were partners/brothers or sisters (G2 generation), and 47% were relatives, friends/other. At the time of the survey, their death occurred 10 ± 7 months ago on average.

### 3.2. Indicators of grief, burden, and wellbeing

In the entire sample, 54% had low psychological wellbeing (WHO-5 score <13), 24% had moderate psychological wellbeing (scores 13–18), and 22% had high psychological wellbeing (scores >18). Regarding grief responses, 15% had no CG response (ICG score <8), 69% had a normal grief response (scores 8–32), and 16% were suspected to have a CG response (scores > 32). Regarding the BGL, 20% had a low grief burden (BGL scores <7), 61% had a moderate grief burden (scores 7–29), and 11% had a high grief burden (scores > 29).

Slight differences were observed in terms of gender for CG, BGL, and perception of burden, but no such differences were observed for psychological wellbeing (Table 2). Regarding the relationship with the deceased person, the loss of a direct partner/brother or sister resulted in very high ICG and low WHO-5 scores as compared to the loss of a parent or relative/others (Table 2). This relational status was not relevant for being BGL or the perception of burden. Furthermore, the place of dying had no significant influence on the indicators of grief, perceived burden, and psychological wellbeing (Table 2). Spiritual/religious self-categorization also had no significant influence (data not shown).

**Table 2:** Indicators of grief, burden, and wellbeing in the study sample.

		**Complicated grief (ICG-D)**	**Burdened by Grief and Loss (BGL)**	**Psychological wellbeing (WHO-5)**	**Perception of burden (5NRS)**
Expected range		0–76	0–36	0–25	0–100
*n*		194	161	161	161
All participants	Mean	21.00	18.27	12.34	43.48
	SD	12.49	10.75	6.11	20.64
**Gender**					
Women	Mean	22.25	19.38	11.92	45.58
	SD	12.12	10.61	5.86	19.48
Men	Mean	16.73	14.12	13.90	35.62
	SD	12.94	10.39	6.85	23.15
*F*-value		6.86	6.65	2.86	6.47
*P*-value		0.010	0.011	n.s.	0.012
Eta^2^ value		0.034	0.040	0.018	0.039
**Relational status**					
Parents (G1)	Mean	20.37	16.22	13.38	42.44
	SD	12.36	10.95	6.71	21.30
Partner/brother or sister (G2)	Mean	32.56	20.48	9.51	44.24
	SD	11.78	12.13	6.04	25.74
Relatives/others	Mean	19.49	19.28	12.46	43.29
	SD	11.40	9.86	5.65	19.48
*F*-value		12.06	1.85	3.47	0.06
*p*-value		<0.001	n.s.	0.034	n.s.
Eta^2^ value		0.142	0.025	0.045	0.001
**Place of dying**					
Hospital	Mean	20.79	19.71	12.77	43.18
	SD	12.68	11.10	6.32	22.22
Nursing home/hospice	Mean	23.04	19.75	11.45	48.60
	SD	15.21	11.39	6.68	19.67
At home	Mean	23.88	14.99	11.39	39.07
	SD	9.93	8.43	5.85	19.70
*F*-value		0.86	2.60	0.86	1.64
*p*-value		n.s.	n.s.	n.s.	n.s.
Eta^2^ value		0.012	0.034	0.012	0.022
**Visits at place were possible**					
No	Mean	22.92	23.65	12.01	46.20
	SD	13.48	9.58	5.91	23.04
Yes	Mean	20.97	15.27	12.50	41.03
	SD	12.15	9.87	6.58	18.76
*F*-value		0.77	24.08	0.20	2.08
*p*-value		n.s.	<0.001	n.s.	n.s.
Eta^2^ value		0.006	0.153	0.002	0.015

Significant differences (p <0.001) were highlighted.

### 3.3. Visits before the death of their relative

Most of the participants had the opportunity to visit their relatives before death (59%), while this was not possible for 41% of the participants. When this was not possible, being BGL was significantly higher (with a strong effect size), while this had no influence on CG, psychological wellbeing, or perceived burden (Table 2).

Furthermore, 43% of the participants stated that they visited their beloved ones in person shortly before they died, and 31% stated that they did not; the remaining participants did not provide any clear information on this. The frequency of these visits was mostly given as once or twice (42%), on average 5 ± 8 times; the duration of these visits was on average 7 ± 13 h.

A significantly large fraction of participants phoned their relatives shortly before their death (33%), while 67% did not and 7% made video calls. Additionally, 46% stated that phoning was not possible, and 5% had not thought of it. Moreover, 50% felt that it was possible to say goodbye to the person before they died, while 50% did not feel so.

The possibility of personal visits on site shortly before the death of the relatives was perceived by most as emotional support for themselves (72%) and helpful for their mourning (75%). Nevertheless, most of them said that they felt emotionally burdened if they had not visited or had only rarely visited the deceased personally on site shortly before their death (75%), and 68% stated missing this in retrospect.

### 3.4. Support satisfaction

Most participants felt that despite the difficult circumstances due to the COVID-19 pandemic-related restrictions, their loved ones received good emotional support/care from the treatment/care team (51%), 22% were undecided, and 27% were negative. Moreover, many participants felt that they, as visitors, were emotionally well-supported/cared for by the treatment/care team (34%), 24% felt that they were partially supported, and 42% felt that they were not supported.

The satisfaction that their beloved ones received good emotional support from the team correlated weakly and inversely with CG scores, correlated moderately and inversely with being BGL, and correlated moderately and positively with psychological wellbeing but not with perceived burden (Table 3). In contrast, feeling well-cared for by the team correlated moderately and inversely with being BGL and with the perception of burden, but it did not correlate significantly with CG or psychological wellbeing. However, none of these indicators of burden correlated significantly with the frequency of visits shortly before death, how long ago their relatives had died (months), or their own age (Table 3).

**Table 3:** Correlation analyses.

	**Complicated grief (ICG-D)**	**Burdened by Grief and Loss (BGL)**	**Psychological wellbeing (WHO-5)**	**Perception of burden (5NRS)**
Complicated grief (ICG-D)	1.000			
Burdened by Grief and Loss (BGL)	0.443^**^	1.000		
Psychological wellbeing (WHO-5)	−0.536^**^	−0.329^**^	1.000	
Perception of burden (5NRS)	0.368^**^	0.278^**^	−0.364^**^	1.000
Perceived relational affection	0.416^**^	0.171	−0.230^**^	0.016
**Possibility of visits**				
Personal visits before death	−0.070	−0.392^**^	0.043	−0.120
Personal visits were emotionally supporting (V1)	−0.135	−0.029	0.124	−0.114
Personal visits shortly were helpful for grieving (V2)	−0.258^**^	−0.173	0.197	−0.114
Visit restrictions were emotionally affecting (V3)	0.338^**^	0.636^**^	−0.174	0.208
Missing the visits that were restricted for mourning (V4)	0.290^**^	0.644^**^	−0.155	0.225
Duration of visits before death (hours)	−0.046	−0.359^**^	0.025	−0.067
Time since relatives had died (months)	−0.026	0.076	0.084	0.023
**Perceived support**				
Relatives were emotionally well-supported by the care team	−0.250^**^	−0.365^**^	0.299^**^	−0.163
Felt that they were well-supported by the care team	−0.149	−0.382^**^	0.184	−0.308^**^
Wished they would have had pastoral accompaniment	0.447^**^	0.332^**^	−0.393^**^	0.235^**^
Wished they would have had psychological accompaniment	0.452^**^	0.288^**^	−0.455^**^	0.183

^**^p <0.001 (Spearman rho); moderate (yellow), and strong correlations (orange) were highlighted.

The support from the treatment/care team was relevant for the visiting relatives. They talked with the treatment/care team about the dying process (29%), talked about farewell and bereavement (19%), and asked organizational questions (33%). Information about pastoral offers or bereavement support was seldom given (12%), and 17% did not want such discussions. Interestingly, 12% reported that they would have liked to receive pastoral support (41% reported they would not have liked it, and 47% were indifferent), and another 12% reported that they would have liked to receive psychological support (49% reported that they would not have liked it, and 39% were indifferent). This wish to receive psychological support/accompaniment was moderately related to CG scores and low psychological wellbeing and weakly related only to the BGL. The intention to receive pastoral support/accompaniment was moderately related to (all of) these indicators of burden (Table 3). Both intentions to receive further support were only weakly related to their satisfaction with the support their relatives or they themselves received (data not shown).

CG scores were moderately related to participants' perceived emotional affections when it was not (or only rarely) possible to visit their relatives in person shortly before their death. They were weakly related to the perception that these visits would have been needed for the mourning process when visits were not possible, and they were similarly related to the perception that these visits would help them grieve (Table 3). Similar associations were not observed for psychological wellbeing or perception of burden (Table 3). However, being BGL was strongly related to perceived emotional affection because visits were restricted, and they missed these visits for mourning; it was moderately related to the possibility of such visits and the duration of visits before death (Table 3). In addition, the BGL was moderately related to satisfaction with the support from the care team and the wish for pastoral accompaniment.

### 3.5. Requirement of professional treatment

Related to the loss and prolonged grief process, 17% of participants (*n* = 27) were in treatment (medical doctor or psychologist/psychotherapist). Furthermore, 52% of those with high ICG scores were in treatment (*n* = 15), and 48% were not (*n* = 14). Among those with moderate ICG scores, only 10% were in treatment (*n* = 11); among those with no CG, only one person was in treatment. These differences were significant (*p* < 0.001, Pearson's Chi-squared test). In contrast, for being BGL, no significant differences in the utilization of professional help (treatment) were observed (data not shown). Of those with high BGL scores (*n* = 28), 89% were not in treatment (*n* = 25).

### 3.6. Predictors of complicated grief and being Burdened by Grief and Loss

As there were several variables with a significant influence on grief reactions, we included these as independent variables in stepwise regression models to predict CG and the Burden of Grief and Loss as dependent variables. As gender had a weak influence, it was added as a confounder.

CG symptoms as a dependent variable were predicted by COVID-19-related burden (explaining 42% of variance), relational status (G2 vs. G1 generation; adding an additional 17% of the explained variance), and (low) psychological wellbeing (adding 10%; Table 4). These three variables together explained 69% of the variance. In this study, the best predictors were relational status and (low) psychological wellbeing.

**Table 4:** Predictors of Complicated Grief and being Burdened by Grief and Loss (stepwise regression analyses).

**Dependent variable: Complicated grief (ICG-D)** ^*^		**Beta**	***T***	***p***
**Model 3:** ***F*** = **21.9**, ***p***<**0.001;** *R*^2^ = **0.69**				
3	(Constant)		0.818	0.420
	Perception of burden (5NRS)	0.353	2.707	0.011
	Relational status (G2 vs. G1)	0.417	4.028	<0.001
	Psychological wellbeing (WHO-5)	−0.402	−3.093	0.004
**Dependent variable: Burdened by Grief and Loss (BGL)** ^**^				
**Model 3:** ***F*** = **16.7**, ***p***<**0.001;** *R*^2^ = **0.63**				
3	(Constant)		−0.226	0.823
	Visit restrictions were emotionally affecting (V3)	0.539	4.701	<0.001
	Relational status (G2 vs. G1)	0.412	3.591	0.001
	Felt well-supported by the care team	−0.319	−2.835	0.008

^*^There was no significant influence in this model by gender, the variables support satisfaction by the team, wishes for pastoral or psychological accompaniment, professional treatment requirement, the possibility of visits and their duration, and the influence of visit restrictions on their emotional situation and grief processes.

^**^There was no significant influence on their grief processes in this model by gender, psychological wellbeing, perception of burden, wishes for pastoral or psychological accompaniment, professional treatment requirement, the possibility of visits, and finally, the influence of visit restrictions.

Being BGL as a dependent variable was best explained by the perception of emotional affection because of restricted visits shortly before death (which explained 37% of the variance). It was also explained by the relational/generational status (G2 vs. G1 generation; adding 16%) and by the (low) perception of being well-supported by the care team (adding an additional 10% of the explained variance; Table 4). These three variables together explained 63% of the variance. In this study, the perception that the visit restrictions emotionally affected the participants had the strongest effect.

Using Cohen's formula to calculate the effect size of the models (*f*^2^ = *R*^2^/1-*R*^2^), for the first model with *R*^2^ = 0.69 and three predictors, we obtained *f*^2^ = 2.33 and, finally, achieved a power of 1.00. For the second model, with *R*^2^ = 0.603 and three predictors, we obtained *f*^2^ = 1.13 and, finally, achieved a power of 1.00. The achieved power confirms that we had the necessary sample size to run the models and predict the reported results.

## 4. Discussion

In the self-selected sample of people who were bereaved during the COVID-19 pandemic, 54% had low psychological wellbeing. Most of them had the opportunity to visit their relatives before death (59%), while 41% reported that this was not possible. In the early stage of the pandemic, several authors (27, 34–36), as well as pertinent organizations (37), expected grief processes to be aggravated due to and during the COVID-19 pandemic and called for more psychosocial and spiritual support for bereaved people and healthcare staff. Our preliminary results (29) showed a pronounced need for pastoral care and support in the mourning process, especially under the additional burdens brought about by the pandemic. They also indicated the lack of available pastoral care workers, psychological support, and low-threshold references by the caring staff of the facilities in the sense of spiritual care.

### 4.1. Differences in grief processes

Regarding grief responses in this sample, 16% were suspected to have a CG response. Regarding being BGL, 11% had a high grief burden. Compared to the German validation study by Lumbeck et al. (31), the mean ICG-D score in our sample was lower by 15 points. In our study, higher ICG-D scores were found when the deceased person was a partner/brother or sister (G2 generation) than when the deceased person was a parent (G1 generation) or a relative. The effect size was strong. This finding is consistent with those of Gang et al. (24). In our study, women had higher CG symptoms than men, while the place of dying or access to visits had no significant influence. This is surprising since one might expect restrictions to impair the process. In addition, while Downar et al. (25) did not find any associations between the severity of grief and physical presence, our study and that of Gang et al. (24) found significant relationships between the two factors. These conflicting findings might become more interesting in light of our additional findings. Specifically, we found that the (non-pathological) Burden of Grief and Loss was not dependent on the relational status or place of dying but on accessibility for visits (with a strong effect size). These two processes appear to be different from each other.

Our initial hypothesis was 2-fold. Specifically, we hypothesized that (1) being BGL is inversely related to both the possibility of visiting the dying relative and the support received from the care team and (2) CG processes depend less on the possibility of visiting the dying relative and the support from the care team as other factors might be more relevant. As stated above, we found that being BGL was influenced more by visit restrictions than CG symptoms and by the support from the care team. The best predictors of CG symptoms were relatives' psychological wellbeing and the relational/generational status (G2 vs. G1), while for being BGL, it was the perception that the visit restrictions were emotionally affecting, as well as the relational/generational status (G2 vs. G1) and the feeling of not being well-supported by the team. More relatives with CG symptoms than those with high BGL scores were receiving psychological treatment, and both groups wished to a similar amount that they would have received psychological and pastoral accompaniment. In other words, CG symptoms were observed more often in relatives with low psychological wellbeing (as either the cause or the outcome), while normal, non-pathological grief processes were more significantly related to the inability to bid farewell because of the restrictions. This was emotionally challenging for them because, as relatives, they missed this for their mourning processes. Visiting relatives perceived their visits as an emotional support for themselves and a source of help for their mourning.

These findings are consistent with the qualitative findings of the “Bereavement Welfare Hub” in London, which highlighted the relevance of good communication with the staff, the relevance of being able to visit the dying relatives in person, the relevance of being supported by the family, and the relevance of receiving bereavement support (26). However, our findings added that relatives' psychological wellbeing (as a precondition) might be crucial to their bereavement process, as well as being overburdened by the pandemic situation. This is also linked to the findings of Chen and Tang (22), which indicated that mental health issues of people bereaved during the pandemic require more attention.

### 4.2. Relevance of staff support

It is important to underline that many participants were satisfied with the emotional support/care from the care team (despite the difficult circumstances due to the COVID-19-related restrictions), both for their dying relatives (51%) and for themselves as visiting relatives (34%). However, a large fraction of them were not satisfied. One might argue that the stressed staff members' primary responsibility was to care for the dying relatives and not for the visitors. Nevertheless, the visiting relatives were also stressed and burdened. Their dissatisfaction, however, hardly led to an explicit wish for further support. Only a few (12%) would have liked to receive additional pastoral or psychological support, and only a small fraction was receiving professional treatment because of their reactions to grief (*n* = 27).

The care/treatment team could be helpful for the bereaved, despite their own emotional and professional burden (12–14). If the staff noticed that the visiting relatives required emotional support and took care of it, the non-pathological grieving process of these relatives might have been better. In this regard, we observed a statistically significant (moderate) inverse association between being BGL and feeling supported by the team, which was not observed for CG. This might be understood in the light of their low wellbeing scores, suggesting that they might have been less susceptible and receptive to emotional/psychological or other support.

### 4.3. Implications for support processes

The aforementioned findings underline the important role of care services for the dying and their relatives. The conversations between team members and relatives addressed not only organizational questions but also the dying process, farewell, and bereavement. Only a small group of the bereaved expressed that they required further support from psychologists or pastoral caregivers, while a significantly large fraction said that they were undecided. Interestingly, the opportunity to receive pastoral offers or bereavement support was rarely mentioned in the talks with the care team. When the patients were deceased, the visiting relatives were no longer in contact with the treatment/care team, and they were left alone in their mourning. They lacked information about who could provide additional support because this was not addressed in the talks with the team. Harrop et al. (38) stated that most bereaved people “had not sought support from bereavement services (...) or their General-Practitioner.” Access to such support is difficult. Therefore, they advised, “increased provision and tailoring of bereavement services, improved information on support options and social/educational initiatives to bolster informal support and ameliorate isolation” (38). The findings from our study support these recommendations.

While Selman et al. (7) mentioned the support of relatives' bereavement processes by hospital clinicians, it is also evident that they had high work stress and burden during the pandemic, and they were dealing with their own fears and worries (10), which frequently resulted in compassion fatigue and burnout (12). For them, their patients were at the forefront of their duties, and the visiting relatives were often an additional burden. Müller et al. (39) proposed an adaptation of the British stepped care model for bereavement (40) as a structural framework to improve bereavement care services. They proposed the first step of basic care provided by the social environment and the second step with two parts: (2a) additional basic support by volunteers who accompany the bereaved persons and (2b) more professional psychoeducation counseling or the activation of resources. The third step then comprised the provision of specific psychotherapy for the mourning process for persons with symptoms of PGD.

During the grieving process, social contact by trusted people (i.e., family) is needed (38), in line with steps 1 and 2a of the aforementioned framework. However, the findings of the present study also underline the important role of highly stressed and burdened healthcare providers in supporting the initial bereavement process. They represent one of the first lines of support; hence, they must be included in the very first step of basic care for bereaved relatives, as already implied in the WHO definition of palliative care.

In future studies, such concrete interventions should be verified. A short screening for the intensity of and potential direction of the grieving processes could be implemented. In this regard, relatives' psychosocial, existential, and spiritual needs could also be assessed (41), as well as the required support provided. Whether such support interventions can prevent prolonged grief processes remains to be shown; at the very least, people at risk could be identified and supported more adequately.

During the pandemic, several barriers were identified, and these included “limited availability, lack of appropriate support, discomfort asking for help and not knowing how to access services” (38). Several of these barriers were already identified before the pandemic, including insensitivity, the absence of anticipated support, poor advice, lack of empathy, and systemic hindrance (42). Already in 2015, Aoun et al. suggested a public health model of bereavement support that categorizes three groups of need and is thus better suited than the former yes/no model (43). For the high-risk (of complex grief issues) group (10%), they recommended referral to mental health professionals; the moderate-risk group (30%) might need some additional support from peers or volunteer-led groups; individuals in the large low-risk group (60%) usually received support from family and friends (43). In most cases, people in the low-risk and moderate-risk groups were satisfied with the support they received, while those in the high-risk group usually considered the received support as inadequate (43). However, the moderate-risk group might pose a challenge, as they need timely support to prevent transitioning to the high-risk group. Qualitative data on bereaved people from Australia showed that “much of this support is provided informally in community settings by a range of people already involved in the everyday lives of those recently bereaved” (44). Empirical data underlined that family, friends, and funeral providers were at the forefront of support, while professionals were less often consulted (44). However, this finding did not argue against their important role; rather, it highlighted that accessibility and work overload remain as problematic issues. Thus, it was underlined that “social models of bereavement care” are needed, specifically those “that fit within a public health approach rather than relying solely on professional care” (44). This has implications for future health care in general and bereavement support, in particular.

### 4.4. Limitations

This study referred to cross-sectional data; thus, no causal interpretations could be drawn, and only associations can be described and interpreted.

Due to the recruitment process, this self-selected sample from one area in Germany with a predominance of Catholics is not representative of the general society. As the participants were mainly reached via the information routes of the diocesan pastoral office and the Palliative Care Forum at Freiburg and further spread via the network of cooperation partners, we may not have reached many individuals who have no association with church-related support centers. Rather, people who were already receiving some support from pastoral care or bereavement counseling primarily participated in this study. In retrospect, with pandemic-related restrictions currently lifted, it becomes important to target more men, as they are usually an understudied group. For future bereavement studies, people with a non-religious background must be addressed. Presumably, they could also benefit from both psychological support and spiritual care or pastoral accompaniment.

The willingness to share personal information about a sensitive topic in this vulnerable group of people was not as high as expected. Furthermore, the high number of people who started the survey but did not proceed with it might indicate that their emotional burden is still high, and thus, they were unable or unwilling to respond to the questions of our study. We cannot exclude the possibility that the perceived burden might have been underrated in our study, and we confirmed that it was important to indicate the possibility of obtaining further help and support.

The findings from mourners in Germany and their grief reactions and related perceptions might not be easily transferable to other cultural contexts. Cultural differences in grief responses were described in other studies (45–47).

## 5. Conclusion

Being able to visit dying relatives shortly before their death was important for relatives' mourning and bereavement processes. This emotional aspect was significantly more relevant to normal, non-pathological grief and loss processes than to CG processes, which are influenced by various other variables, including psychological stability. This underlines that there are significant clinical differences between more or less normal, non-pathological mourning processes and complicated or prolonged grief.

The support from their dying relatives' treatment team was highly relevant for the mourning process, but the visiting relatives often lacked information about or had little access to additional resources such as psychologists or pastoral care professionals. Opportunities to obtain pastoral offers or other bereavement support were rarely mentioned by the caring team. This should and can be easily improved at a fundamental level by providing basic information to also care for mourning relatives; otherwise, they may be left alone when they cannot rely on the support of other family or community members. Although direct social contacts (including family members) are highly important for the mourning process, these contacts were also restricted. In Harrop et al.'s (38) study, 39% had problems receiving the required support from family/friends. Thus, it is evident that either additional routes of support are needed, or that the already established support services must be better anchored in the healthcare provider's recommendation paths and in the public consciousness.

Finally, we can learn the following from the outcomes of the pandemic-related restrictions: While these measures were important to protect vulnerable groups, they simultaneously led to the isolation of both the vulnerable people when they were dying and their relatives as well.

## Data availability statement

According to the data protection regulations, the data set cannot be made publicly available. Data are however available from the first author upon reasonable request.

## Ethics statement

The studies were conducted in accordance with the local legislation and institutional requirements. The participants provided their written informed consent to participate in this study.

## Author contributions

AB and KB designed the study and wrote the manuscript. AB set up the online survey and undertook the statistical analyses. All authors approved the final manuscript.
